# Intensity-modulated radiotherapy has superior outcomes to three-dimensional conformal radiotherapy in patients with stage IE-IIE extranodal nasal-type natural killer/T-cell lymphoma

**DOI:** 10.18632/oncotarget.16138

**Published:** 2017-03-11

**Authors:** Yi-Yang Li, Hai-Qun Lin, Lu-Lu Zhang, Ling-Ling Feng, Shao-Qing Niu, Han-Yu Wang, Yu-Jing Zhang, Xi-Cheng Wang

**Affiliations:** ^1^ Department of Oncology, First Affiliated Hospital of Guangdong Pharmaceutical University, Guangdong, People's Republic of China; ^2^ Department of Radiation Oncology, Sun Yat-Sen University Cancer Center, State Key Laboratory of Oncology in South China, Collaborative Innovation Center for Cancer Medicine, Guangzhou, Guangdong, People's Republic of China; ^3^ Department of Radiation Oncology, Shandong Cancer Hospital and Institute, Shandong University School of Medicine, Shandong, People's Republic of China; ^4^ Department of Oncology, Seventh Affiliated Hospital of Sun Yat-Sen University, Shenzhen, Guangdong, People's Republic of China; ^5^ Department of Radiation Oncology, First Affiliated Hospital of Sun Yat-Sen University, Guangzhou, Guangdong, People's Republic of China

**Keywords:** extranodal natural killer/T-cell lymphoma, three-dimensional conformal radiotherapy, intensity-modulated radiotherapy

## Abstract

We compared the treatment outcomes, toxicities and prognoses of patients with stage IE-IIE extranodal natural killer/T-cell lymphoma (ENKTL) treated with intensity-modulated radiotherapy (IMRT) or three-dimensional conformal radiotherapy (3DCRT). Newly diagnosed early-stage ENKTL patients (*N* = 173) were enrolled and received extended involved-field radiotherapy following induction chemotherapy. Patients were treated with 3DCRT (*n* = 98) or IMRT (*n* = 75). One-to-one matching of the IMRT and 3DCRT groups was performed through propensity score matching, which yielded 23 pairs of patients. The two groups achieved similar complete remission rates before and after radiotherapy (*P* > 0.05). All patients were followed up for a median of 41 months. The rates of local recurrence-free survival (LRFS, *P* < 0.001), progression-free survival (PFS, *P* = 0.003) and overall survival (OS, *P* = 0.003) were longer in the IMRT than 3DCRT group. In the matched patients, IMRT was still associated with superior LRFS (*P* = 0.024), but not with improved PFS (*P* = 0.113) or OS (*P* = 0.115). Multivariate analysis also suggested IMRT was a favorable independent factor for LRFS (HR = 2.230, *P* = 0.043), but not for PFS (*P* = 0.195) or OS (*P* = 0.116). Equivalent acute toxicities were observed for 3DCRT and IMRT; however, among stage II patients who had received cervical irradiation, the rate of late xerostomia was lower in the IMRT than 3DCRT group (38.5% *vs*. 66.7%, *P* = 0.046). Overall, IMRT yielded a better treatment response and local control than 3DCRT, and tended to reduce late xerostomia in patients with cervical irradiation, but failed to enhance OS. Thus, IMRT is recommended for the treatment of stage IE-IIE ENKTL patients.

## INTRODUCTION

Extranodal natural killer/T-cell lymphoma (ENKTL) is a distinct clinicopathologic entity with invasive behavior [1–2]. ENKTL is prevalent in Asian countries, and usually arises in portions of the upper aerodigestive tract, such as the nasal cavity or nasopharynx [3–7]. Although early-stage ENKTL represents 70-90% of cases, the clinical management of early-stage ENKTL remains controversial. Nevertheless, radiotherapy (RT) is well acknowledged as the primary therapy for early-stage ENKTL patients [8–10].

As techniques have developed, conformal RTs such as intensity-modulated radiotherapy (IMRT) and three-dimensional conformal radiotherapy (3DCRT) have replaced conventional two-dimensional radiotherapy (2DRT), and have been widely applied in clinical practice [11–18]. In previous studies, the four-year loco-regional control exceeded 80% in patients treated with IMRT or 3DCRT, and no severe toxicity occurred [19–21]. In fact, because IMRT is an intensity-modulated technique, it has typically provided better dose coverage of organs at risk (OARs) than 3DCRT. According to Shen et al., IMRT was associated with a significantly higher conformal index (1.28 vs. 1.08) and homogeneity index (0.2 vs. 0.1) than 3DCRT (20). Owing to its dosimetric advantage, IMRT significantly reduced the rate of parotid gland hypofunction and improved the quality of life.

However, few studies have focused on the survival differences of ENKTL patients treated with IMRT and 3DCRT. To determine the differences between IMRT and 3DCRT, we analyzed clinical data from a large cohort of early-stage ENKTL patients treated with IMRT or 3DCRT, performed propensity score matching (PSM) to balance the groups in terms of their chemotherapy regimens, and compared the groups for their radiation-induced toxicity and survival.

## RESULTS

### Clinical characteristics

Among the cohort, 140 patients presented with ENKTL from the nasal cavity, 26 from Waldeyer's ring, and 7 from other parts of the upper aerodigestive tract, such as the hard plate, buccal cavity and gingiva. The median patient age was 40 years (range, 11-75). Of the patients, 109 were diagnosed with stage I and 64 were diagnosed with stage II ENKTL. The male/female ratio was 1.9/1 (114/59). B symptoms and elevated lactate dehydrogenase (LDH) levels were observed in 68 and 34 patients, respectively. The numbers of patients with stage-modified International Prognostic Index (mIPI) values of 0 to 4 were 45, 52, 55 and 21.

The baseline clinical characteristics of the IMRT and 3D-CRT groups are listed in Table 1. A higher proportion of patients had received asparaginase-based chemotherapy in the IMRT group than in the 3DCRT group (58.7% for the IMRT group vs. 12.2% for the 3DCRT group, P = 0.001). In addition, the IMRT group tended to have higher mIPI scores than the 3DCRT group. To exclude the effects of confounding factors, we performed PSM and set the caliper score as 0.002. Ultimately, we obtained 23 pairs of patients. After PSM, all baseline clinical characteristics became comparable in the IMRT and 3D-CRT groups (Table 1).

**Table 1:** Patient characteristics of all patients

Characteristics	Before PSM			After PSM		
	IMRT (*n*=75,%)	3DCRT (*n*=98,%)	*P* value	3DCRT (*n*=23,%)	IMRT (*n*=23,%)	*P* value
Sex			0.152			0.475
male	45(60)	69(70.4)		19(82.6)	17(73.9)	
female	30(40)	29(29.6)		4(17.4)	6(26.1)	
Stage			0.813			0.765
I	48(64)	61(62.2)		14(60.9)	13(56.5)	
II	27(36)	37(37.8)		9(39.1)	10(43.5)	
Age			0.423			1.000
≤60	68(90.7)	85(86.7)		20(87)	20(87)	
>60	7(9.3)	13(13.3)		3(13)	3(13)	
B symptoms			0.436			0.697
present	27(36)	41(41.8)		5(21.7)	3(13)	
absent	48(64)	57(58.2)		18(78.3)	20(87)	
Primary site			0.584			1.000
Nasal cavity	59(78.7)	81(82.7)		19(82.6)	20(87)	
Waldeyering's ring	13(17.3)	13(13.3)		4(17.4)	3(13)	
Hard plat	2(2.7)	2(2.0)		0	0	
buccal cavity	1(1.3)	1(1.0)		0	0	
gingiva	0	1(1.0)		0	0	
ELTI			0.315			0.300
absent	34(45.3)	37 (37.8)		7(30.4)	4(17.4)	
present	41(54.7)	61 (62.2)		16(69.6)	19(82.6)	
LDH level			0.465			1.000
elevated	17(22.7)	17(17.3)		3(13)	2(8.7)	
normal	58(77.3)	81(82.7)		20(87)	21(91.3)	
PET/CT			0.001			0.359
absent	27(36)	85(86.7)		13(56.5)	16(69.6)	
present	48(64)	13(13.3)		10(43.5)	7(30.4)	
Induction chemotherapy			0.001			0.369
Asparaginase-based	44(58.7)	12(12.2)		8(34.8)	11(47.8)	
asparginase-absent	31(41.3)	86(87.8)		15(65.2)	12(52.2)	
Response to chemotherapy			0.226			1.000
CR	32(42.7)	33(33.3)		10(43.5)	10(43.5)	
Non-CR	43(57.3)	65(66.7)		13(56.5)	13(56.5)	
mIPI			0.001			0.653
0	22(29.3)	23(23.5)		4(17.4)	3(20)	
1	21(28.0)	31(31.6)		10(43.5)	8(26.7)	
2	23(30.7)	32(32.7)		5(21.7)	10(36.7)	
3	9(12.0)	12(12.2)		4(17.4)	2(16.7)	

Abbreviations: ELTI, extensive local tumor invasion; LDH, lactate dehydrogenase; CR, complete response; mIPI, stage-modified International Prognostic Index

### Treatment outcomes

**Figure 1:**
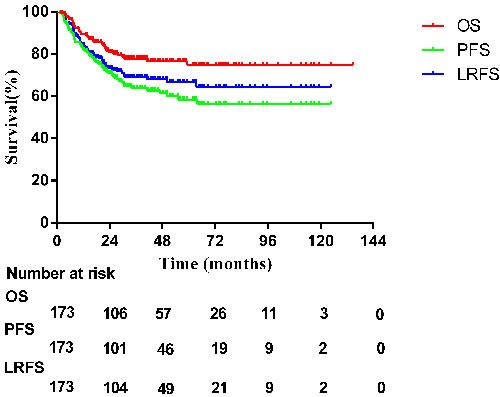
Survival curves for all patients

**Figure 2:**
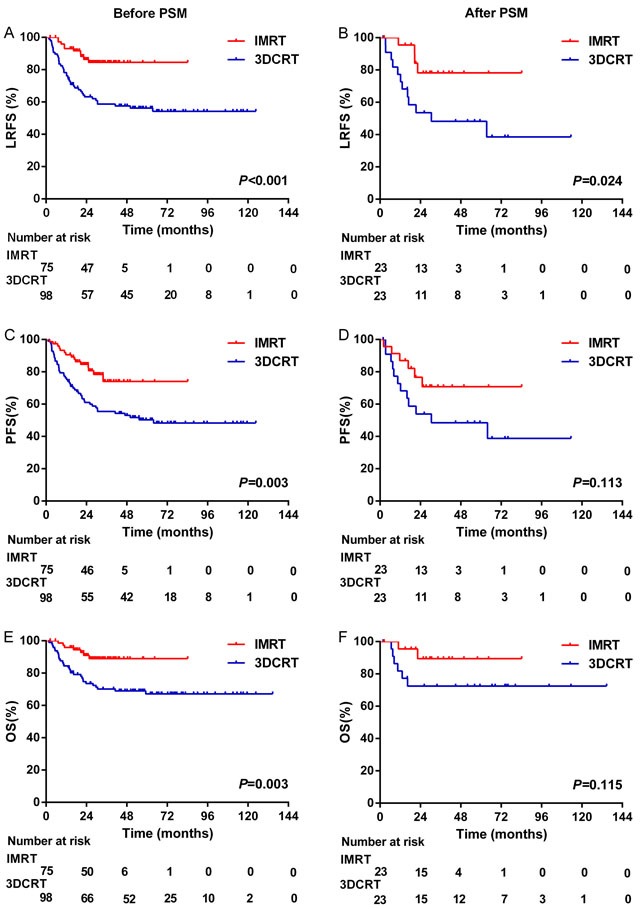
Local recurrence-free survival, progression-free survival, and overall survival for patients before and after propensity score matching

Thirty-three patients from the 3DCRT group and 32 from the IMRT group experienced complete remission (CR) after chemotherapy (33.3% vs. 42.7%, *P* = 0.226). After radiotherapy, 83.8% of patients (109/173) achieved CR. Similar CR rates were observed in the IMRT group (52/75, 69.3%) and the 3DCRT group (57/98, 58.2%, *P* = 0.132). By the end of May 2016, all patients had been followed up for 2-135 months. The median follow-up time for surviving patients was 28 months in the IMRT group and 52 months in the 3DCRT group. Overall, patients achieved three-year local recurrence-free survival (LRFS), progression-free survival (PFS) and overall survival (OS) rates of 75.3%, 69.0% and 81.3%, respectively (Figure 1). However, subgroup analysis demonstrated that the LRFS, PFS and OS rates in the IMRT group were superior to those in the 3DCRT group (*P* < 0.001), as shown in Figure 2.

After PSM, both groups exhibited similar CR rates before (43.5% for both the IMRT group and the 3DCRT group, *P* = 1.000) and after radiotherapy (65.2% for the IMRT group vs. 56.5% for the 3DCRT group, *P* = 0.546). IMRT demonstrated better local control than 3DCRT in terms of LRFS (*P* = 0.024). However, this did not ultimately result in significant differences in PFS or OS between the groups (*P* = 0.113 and 0.115, respectively; Figure 2)

### Prognostic analysis

The variables in Table 1 were included in the univariate analysis. Age, response to chemotherapy, RT technique, and extensive local tumor invasion (ELTI) were identified as significant prognostic factors of OS, PFS and LRFS in univariate analysis (Table 2). When these factors were taken into account, multivariate analysis revealed that IMRT was an independent factor correlated with superior LRFS (HR = 2.230, 95%CI = 1.027-4.845, *P* = 0.043), but was not an independent prognostic factor for PFS (*P* = 0.195) or OS (*P* = 0.116). In addition, CR after chemotherapy was an independent favorable factor for LRFS and PFS (*P* = 0.018 and 0.019, respectively), and tended to be associated with improved OS (*P* = 0.091). Asparaginase-based chemotherapy was another favorable prognostic factor for PFS (*P* = 0.045), while an age >60 (*P* = 0.012) and ELTI (*P* = 0.028) were independent negative prognostic factors for OS.

**Table 2:** Univariate analysis for LRFS, PFS, and OS

	3-year LRFS		3-year PFS		3-year OS		
	%	*P* value	%	*P* value	%	*P* value
Sex		0.404		0.721		0.311
male	66.3		62.9		76.2	
female	75.0		68.6		80.7	
Age		0.014		0.009		0.001
>60	41.2		35.4		44.9	
≤60	73.0		67.8		82.2	
Stage		0.167		0.169		0.218
I	73.0		67.7		80.7	
II	62.6		57.2		73.2	
B symptom		0.889		0.976		0.592
present	70.1		65		82.9	
absent	68.6		63.1		74.3	
Primary site		0.330		0.218		0.802
Nasal cavity	70.3		65.7		77.4	
Waldeyering's ring	62.2		51.9		80.2	
ELTI		0.066		0.022		0.006
absent	75.8		72.7		88.1	
present	64.4		57.4		70.3	
LDH level		0.490		0.270		0.299
elevated	65.7		57		71.3	
normal	70.0		65.4		79.4	
PET		0.291		0.328		0.622
Absent	66.2		61.5		76.5	
Present	75.9		67.7		80.8	
RT technique		0.001		0.003		0.003
IMRT	84.5		73.9		88.9	
3DCRT	58.7		55.4		70.1	
Response to chemotherapy		0.002		0.001		0.014
CR	84.2		79.2		87.8	
Non-CR	60.6		54.8		72.1	
Chemotherapy regimen		0.001		0.001		0.006
Asparaginase-based	87.9		78.5		91.8	
asparginase-absent	61.3		56.2		71.9	

Abbreviations: ELTI, extensive local tumor invasion; LDH, lactate dehydrogenase; RT, radiotherapy; CR, complete response; mIPI, stage-modified International Prognostic Index; LRFS, local recurrence-free survival; PFS, progression free survival; OS, overall survival.

### Acute and late toxicities

Data related to acute radiation-induced toxicity are shown in Table 4. No patients ceased treatment due to severe toxicity. The most common acute toxicity was mucositis (172/175, 98.3%), which was scored as Grade 1 or 2 in 159 patients, and as Grade 3 or 4 in 13 patients. The overall rate of acute toxicities did not differ significantly between the 3D-CRT and IMRT groups. Late toxicity data at one-year follow-up could only be recorded in 149 patients - 70 from the IMRT group and 79 from the 3DCRT group. The most common late toxicity, xerostomia, was observed in 30 patients. Among them, 18/79 patients (22.8%) in the 3D-CRT group and 12/70 (17.1%) in the IMRT group presented with Grade 1 or 2 xerostomia; this difference was statistically insignificant (*P* = 0.391). No patients experienced Grade 3 or 4 xerostomia. However, among patients with stage II ENKTL, a lower rate of xerostomia was detected in the IMRT group than in the 3DCRT group (10/26, 38.5% for IMRT vs. 16/24, 66.7% for 3DCRT, *P* = 0.046). No other serious late toxicities were recorded.

**Table 3:** Multivariate analysis for LRFS, PFS, and OS

	LRFS		PFS		OS	
	HR(95%CI)	*P*	HR(95%CI)	*P*	HR(95%CI)	*P*
Age >60	1.858(0.942-3.667)	0.074	1.845(0.986-3.450)	0.055	2.595(1.233-5.461)	0.012
ELTI	1.467(0.810-2.655)	0.206	1.715(0.987-2.978)	0.056	2.426(1.102-5.342)	0.028
3DCRT	2.230(1.027-4.845)	0.043	1.544(0.800-2.981)	0.195	2.104(0.832-5.323)	0.116
CR after chemotherapy	0.430(0.214-0.866)	0.018	0.475(0.255-0.885)	0.019	0.486(0.210-1.036)	0.091
Asparaginase-based chemotherapy	0.448(0.172-1.169)	0.101	0.424(0.184-0.981)	0.045	0.440(0.136-1.424)	0.171

Abbreviations: ELTI, extensive local tumor invasion; 3DCRT, three-dimensional conformal radiotherapy; CR, complete response; LRFS, local recurrence-free survival; PFS, progression free survival; OS, overall survival

**Table 4:** Acute radiation toxicities in all patients

	Grade1+2		*P*	Grade3+4		*P*
	3DCRT(98)	IMRT(75)		3DCRT(98)	IMRT(75)	
Xerostomia	80(81.6%)	64(85.3%)	0.518	0	0	1
Dysphagia	57(58.2%)	43(57.3%)	0.913	1(1%)	1(1.3%)	0.849
Mucositis	90(91.8%)	69(92.0%)	0.969	7(7.1%)	6(8.0%)	0.832
Skin reaction	93(94.9%)	72(96%)	0.732	3(3.1%)	2(2.7%)	0.878

### DISCUSSION

As conformal radiotherapies, IMRT and 3DCRT each have particular advantages [9]. Compared with IMRT, 3DCRT is cheaper and easier to implement. However, IMRT is usually superior to 3DCRT in its dose coverage, meaning that it has a better dose conformity index and homogeneity index [20, 21]. In our study, IMRT was superior to 3DCRT in its effects on the LRFS, PFS, and OS rates (*P* < 0.001). However, when the effects of confounding factors were eliminated with PSM, the OS benefit was no longer apparent, suggesting that IMRT was only associated with better local control in ENKTL patients. This could be attributed to the dose advantage of IMRT [20, 21]. However, according to Shen et al., the dose advantage of IMRT in ENKTL patients did not include a survival benefit in terms of four-year OS (80.9% for 3DCRT vs. 82.7% for IMRT, *P* = 0.87) or four-year LCP (86.3% for 3DCRT vs. 88.9% for IMRT, *P* = 0.85) [20]. This could be explained by the unbalanced tumor responses to chemotherapy regimens between IMRT group and 3DCRT group in Shen's study. In the current study, all patients received induction chemotherapy and presented similar responses to chemotherapy after PSM, and IMRT was ultimately established as an independent factor associated with improved LRFS. As a result, we suggest IMRT instead of 3DCRT as the standard radiotherapy treatment for early-stage ENKTL.

In addition to survival, the protection of OARs was also emphasized. Even with superior dose coverage, IMRT was equivalent to 3DCRT in its average mean dose to OARs [20]. We compared the acute and late toxicities of both treatments, and detected no significant differences between IMRT and 3DCRT. However, subgroup analysis indicated that the rate of xerostomia for stage II patients with cervical lymphoid region irradiation was lower in the IMRT group than in the 3DCRT group. Consistent with the results of the current study, Shen et al. reported that IMRT resulted in a lower mean dose to the parotid gland than 3DCRT in stage II patients receiving cervical irradiation [20]. Due to its better parotid gland avoidance, IMRT reduced the incidence of xerostomia, which mainly benefited stage II ENKTL patients.

Regardless of improved survival and toxicity, the quality of life after treatment is important. Although no published studies have focused on the quality of life in ENKTL patients, data from nasopharyngeal carcinoma patients revealed that IMRT improved the quality of life after radiotherapy [22]. We are collecting data on life quality among ENKTL patients, and expect to find promising results. Overall, ENKTL patients can benefit from the superior treatment outcomes and reduced toxicity of IMRT.

In the era of IMRT, the accurate delineation of target volume is of vital importance. Recently, positron emission tomography-computed tomography (PET-CT) has emerged as a useful staging and diagnostic tool [23]. PET-CT is also important for treatment planning, as it can provide biological information on tumor invasion and can identify uncertain invasion sites detected by magnetic resonance imaging (MRI) or CT, ultimately aiding in the precise delineation of the target volume. In patients who do not undergo PET-CT, the target volume tends to include more regions, as a means of ensuring that all possible involved sites and uncertain inflamed mucosa are irradiated. In such cases, severe toxicity may occur, which could otherwise have been lessened or avoided. Pretreatment PET-CT is still preferred to achieve an accurate radiation plan.

Although ENKTL is sensitive to radiation, chemotherapy is still necessary to reduce the chance of distant metastasis. The treatment outcome of induction chemotherapy was found to be associated with the final treatment outcome in early-stage ENKTL patients [24]. In the present study, a significant survival advantage was observed in patients achieving CR after induction chemotherapy. Many studies have proposed asparaginase-based chemotherapy for the treatment of ENKTL, as it has demonstrated dramatic advantages over conventional anthracycline-based regimens [25–27]. Our previous data demonstrated that patients treated with GELOX chemotherapy (gemcitabine, L-asparaginase and oxaliplatin) followed by IMRT reached a two-year OS and PFS of 86% [24]. Thus, the combination of asparaginase-based chemotherapy and IMRT can greatly improve patients' final outcomes and quality of life, making it the optimal choice for early-stage ENKTL patients.

There are limitations to the current study due to its retrospective nature. First, the baseline characteristics of the two groups were unbalanced. To exclude the bias from these factors, we employed PSM in statistical analysis. Even so, chemotherapy heterogeneity was present. Various chemotherapy regimens were used in our study, such as CHOP, EPOCH, ATT and DEVIC for asparaginase-absent regimens, and P-Gemox and CHOP-L for asparaginase-based regimens (see Materials and Methods). However, considering the more important status of radiotherapy in early-stage ENKTL, we think that the influence of the various regimens was insignificant. Besides, the quality of the radiation plans from different doctors and the prescribed doses to the target volume varied from person to person, which may have contributed to the discrepancies between IMRT and 3DCRT to some extent. Thus, much prospective work needs to be done to reduce these confounders. Another problem was that after PSM, the number of patients (46 of 173) was too small for outcomes to be compared. However, considering the rarity of ENKTL, we think that this small deficit is acceptable and that the conclusions of current study will still be valuable in guiding clinical practice [2–3].

Compared with 3DCRT, IMRT seems to produce an increased treatment response and improved local control, though it does not seem to enhance OS. Although IMRT displayed no significant advantage over 3DCRT in its acute toxicity, it did cause a lower rate of xerostomia in patients with cervical irradiation. Thus, IMRT is recommended for the treatment of stage IE-IIE ENKTL patients.

## MATERIALS AND METHODS

### Patient characteristics

Between 2003 and 2013, a total of 173 ENKTL patients (stage I-II) were admitted to Sun Yat-Sen University Cancer Center and underwent definitive conformal radiotherapy (IMRT or 3DCRT). All these patients were diagnosed with typical morphology and immunohistochemistry in accordance with the World Health Organization classification of lymphoid neoplasms [1]. Informed consent for the collection of medical information was obtained from each patient at the first visit, and the ethics committee of Sun Yat-Sen University Cancer Center approved this study.

Among these patients, 140 presented with ENKTL from the nasal cavity, 26 from Waldeyer's ring, and 7 from other parts of the upper aerodigestive tract, such as the hard plate, buccal cavity and gingiva. Before treatment, patients received a complete medical history evaluation, a thorough physical examination, a series of laboratory tests, and complete imaging materials, mainly including head and neck MRI and CT scans of the chest, abdomen, and pelvis. Certain patients also underwent PET-CT for the detection of lymphomas. In reference to these examinations, ENKTL were staged in accordance with the Ann Arbor system. The mIPI was adopted to predict prognosis [22], in which a score of 1 was given for each adverse prognostic factor, including an age >60, Ann Arbor stage II, elevated LDH level, >1 extranodal site involved, and Eastern Cooperative Oncology Group performance score >1. ELTI was defined as occurring when tumors spanned neighboring structures (such as the nasal skin, paranasal sinus, orbit, and hard or soft palate) by contiguous spread. B symptoms were defined as unexplained recurrent fevers (temperatures above 38°C), night sweats, and unexplained weight loss of more than 10% in the six months before diagnosis.

### Treatment

Before definitive radiotherapy, all patients were initially treated with one to six cycles of induction chemotherapy. The prescribed regimens varied among different physicians. In the early years, asparaginase-absent regimens were the most common chemotherapy regimens used for ENKTL patients. However, with the development of chemotherapy since 2007, these have gradually been replaced by asparaginase-based regimens. In sum, 117 patients received asparaginase-absent chemotherapy, while 56 were treated with asparaginase-based regimens. In the current study, asparaginase-absent regimens included CHOP (vincristine, doxorubicin, cyclophosphamide, prednisone, 30/117, 25.6%), EPOCH (etoposide, vincristine, doxorubicin, cyclophosphamide, prednisone, 53/117, 45.3%), DEVIC (etoposide, carboplatin, cyclophosphamide, dexamethasone, 3/117, 2.6%), and ATT (altering triple therapy, 31/117, 26.5%) with CHOP-B (vincristine, doxorubicin, cyclophosphamide, prednisone, bleomycin), IMVP-16 (ifosfamide, methotrexate, etoposide, prednisone) and DHAP (dexamethasone, cisplatin, cytarabine). As for the asparaginase-based regimens, the most commonly used regimen was P-Gemox (gemcitabine, pegasparagase, oxaliplatin, 51/55, 92.7%), while CHOP-L (vincristine, doxorubicin, cyclophosphamide, prednisone, L- asparaginase) was administered to 4/55 patients (7.3%). All these regimens were administered at three weeks per cycle.

Following chemotherapy, 75 patients received IMRT and 98 received 3DCRT. While immobilized in a supine position with a perforated thermoplastic head mask, each patient received a 3-mm slice-thickness CT scan from the vertex of the skull to the inferior of the clavicular heads. Based on the pretreatment PET-CT, MRI and nasopharyngoscopy findings, the gross tumor volume was delineated on the treatment-planning CT images. Different clinical tumor volumes (CTVs) were set according to the risk of the tumor invading adjacent structures. In cases originating from the nasal cavity, a high-risk clinical tumor volume (CTV1) consisted of the entire nasal cavity mucosa, half of the ipsilateral maxillary sinus, the bilateral partial ethmoid sinus and sphenoid sinus, and the hard palate. A low-risk clinical tumor volume (CTV2) was outlined on the basis of CTV1 expansion, and included the whole ipsilateral maxillary sinus and half of the contralateral maxillary sinus. However, if the maxillary sinus was obviously invaded, the whole ipsilateral maxillary sinus was included in CTV1. When both the nasal cavity and the nasopharynx were invaded, Waldeyer's ring was included in CTV1 and the upper cervical region was covered in CTV2. If cervical lymph nodes were found to be involved, CTV2 tended to include the cervical region. However, cervical lymph nodes were not irradiated for patients with stage IE ENKTL. When the primary tumor was located in the nasopharynx, the whole Waldeyer's ring tended to be included in CTV1, and the partial nasal cavity next to the nasopharynx was included in CTV2. The doses were prescribed by different radiation oncologists, ranging from 40-66 Gy, with 2-2.3 Gy/fraction/day and five days/week. The doses to OARs were constrained as follows: maximum dose (D_max_) to brainstem < 50 Gy; D_max_ to spinal cord < 45 Gy; D_max_ of lens < 10 Gy; mean dose (D_mean_) of parotid < 26 Gy. All IMRT treatment plans were generated through an inverse planning system. One anterior portal and two lateral fields were set in 3DCRT plans, and additional electron beams were used to compensate for the insufficient dose to the anterior ethmoid sinus.

### Follow-up

Radiation-induced toxicities were recorded and graded according to the Radiation Therapy Oncology Group and the European Organization for Research and Treatment of Cancer radiation morbidity scoring criteria [23]. Based on the Revised Response Criteria of Malignant Lymphoma, tumor responses were assessed through physical examinations, nasopharyngoscopy and MRI or PET-CT every two cycles during chemotherapy and one month after RT [24]. Patients were then followed up in an out-patient clinic or by phone. Any local recurrences or distant metastases were confirmed by imaging or biopsy. OS was defined as the interval from the time of treatment to the time of death from any cause, or to the time of the last visit. PFS was measured from the date of treatment to the date of the first documented recurrence and/or distant metastasis, or to the date of the last follow-up visit. LRFS was calculated from the initiation of treatment to the patient's local recurrence, death, or last visit.

### Statistical analysis

The baseline characteristics of the IMRT and 3DCRT groups were compared through χ^2^ analysis. OS, PFS and LRFS curves were obtained by the Kaplan-Meier method and compared by the log-rank test. All identified variables in univariate analysis were included in multivariate analysis involving a Cox proportional hazards regression model. To balance the selective bias from chemotherapy regimens and other baseline characteristics between the IMRT and 3DCRT groups, we performed one-to-one matching through PSM analysis according to age, stage, B symptoms, primary site, LDH level, chemotherapy regimen, and CR after chemotherapy. A two-sided *P* value < 0.05 was considered statistically significant. SPSS 19.0 software was used for the statistical analysis.
